# Drivers and Dynamics of Collaborative Governance in Environmental Management

**DOI:** 10.1007/s00267-022-01769-7

**Published:** 2023-02-10

**Authors:** Nicola Ulibarri, Mark T. Imperial, Saba Siddiki, Hayley Henderson

**Affiliations:** 1 Department of Urban Planning & Public Policy, University of California Irvine, Irvine, CA USA; 2 Department of Public & International Affairs, University of North Carolina Wilmington, Wilmington, NC USA; 3 Maxwell School of Citizenship & Public Affairs, Syracuse University, Syracuse, NY USA; 4 Crawford School of Public Policy, Australian National University, Canberra, ACT Australia

**Keywords:** Collaborative governance, Developmental dynamics, Outputs and outcomes, Accountability

## Abstract

This special issue brings together new case studies and comparative works highlighting the drivers and dynamics of collaborative environmental governance. Each case is part of the Collaborative Governance Case Database, which is an open-access resource allowing individuals to contribute and access cases to support research projects. This article highlights the special issue’s contributions to collaborative governance theory. Common themes that cut across the studies include: the importance of using a broad definition of collaborative governance to capture the diversity in interorganizational relationships across contexts; improving our understanding of the drivers for initiating collaborative governance; an enhanced understanding collaboration’s lifecycle dynamics and developmental trajectories; the importance of individuals and their roles in collaborative processes; the political dynamics of collaboration; the role of accountability; and the challenges associated with assessing the performance of collaborations. Collectively, the cases also demonstrate the value of using resources such as the Collaborative Governance Case Database to undertake small-n and medium-n comparative studies that further theory building.

## Introduction

Collaboration is a prominent feature of environmental management. Environmental problems are complex, marked by uncertainties about their causes and consequences (Ulibarri 2019) and often containing multiple interacting feedback loops (Brogden 2003). The capacity for addressing environmental problems (e.g., leadership, staff, legal authority, technology, funding, etc.) is dispersed among actors at different levels of government and different types of organizations, none of which can solve problems by acting alone (Bressers et al. 1994). And the timescale on which environmental changes are visible are often much longer than human management cycles (Thomas and Koontz 2011). In such settings, collaborative governance can support collective decision making, help stakeholders develop shared policies or priorities, improve coordination among relevant stakeholders, and find ways for stakeholders to work productively together (Emerson and Nabatchi 2015a; Imperial 2005a; Milward and Provan 2000; Wood and Gray 1991).

This special issue includes a collection of papers that address the strengths and limitations of collaborative governance to respond to the unique “challenges posed by the dynamism and uncertainties associated with environmental change” (Emerson and Gerlak 2014, 768). In particular, the papers focus on the creation and longer-term evolution of environmental collaborative governance regimes (CGRs). When viewed over longer periods of time, it is increasingly evident that collaborative governance is dynamic (Imperial et al. 2016). CGRs ebb and flow, or become dormant or extinct, only to resurface with new members, names, forms, or boundaries (Genskow and Born 2006). Understanding the drivers for initiating CGRs and how they develop over time is critical to building theory about how collaborations respond to and manage environmental problems. However, much existing knowledge about the drivers, dynamics, and outcomes of collaborative governance stems from single case studies in a relatively small subset of countries and political systems.

Recognizing the potential benefits of large(r)-N studies of collaborative governance, teams of scholars are developing repositories of collaboration cases that can be widely accessed to support cross-case analyses. One prominent repository is the Collaborative Governance Case Database (https://collaborativegovernancecasedatabase.sites.uu.nl/). The Collaborative Governance Case Database (hereafter, “Database”) was first initiated as part of the Successful Public Governance Research Program at Utrecht University that seeks to advance an understanding of successful public policies, organizations, and collaborations across sectors and geographies (Douglas et al. 2020). The Database is a collective, open-access resource that allows individuals to contribute and access cases to support research projects.

Cases are coded into the Database using a code form to facilitate cross-case analysis. The code form contains questions relating to different dimensions of collaborative cases, including the starting conditions of the collaboration, institutional design, leadership, collaborative process, accountability, and outcomes and outputs of the collaboration. The code form prompts researchers to code (where possible) for selected dimensions at different points in time (at the start, middle, and end of the period observed). The code form also asks researchers to input information in response to open-ended questions as well as questions/statements with Likert scales. By presenting cases in a systematic format, the CG database enables both small-n and especially medium-n comparative case studies with data analysis methods (e.g., Qualitative Comparative Analysis). This enables the identification of complex forms of causation and more generalizable^1^ conclusions relating to collaborative governance. For example, Ansell et al. (2020) used 39 cases from different contexts and policy areas to evaluate factors that affect inclusion in collaboration.

However, many of the original cases in the Database demonstrate a tendency for collaborative governance research to focus on certain geographies and sectors. Indeed, much theoretical and empirical work on collaborative governance draws on cases from North America or Northern or Western Europe—liberal, democratic, developed societies—and from a handful of sectors like water governance and urban policy. We know from many disciplines relevant to environmental management, like in ecological studies, urban ecology and urban biodiversity, there is an overwhelming dominance of Global North research published (in some instances up to 90% of publications), meaning that research does not account for the unique dynamics of Global South contexts where most people live and where most environmental challenges are faced (Shackleton et al. 2021). The critique applies to studies of collaborative environmental governance: by overlooking collaborations in other political, socioeconomic, and environmental contexts, the field is missing important nuance on where and how collaboration works.

Responding to the opportunity presented by the Database, this Special Issue brings together new case studies and comparative works that consider collaboration in environmental management across a diversity in contexts. In utilizing the Database, and while acknowledging more work is needed to continue to broaden and diversify contributions to theory, the objectives of this special issue are to:

- Generate a set of comparable case studies for inclusion in the collaborative case database.
- Expand an understanding of the drivers and dynamics of collaborative governance in lessor studied political, geographic, and environmental contexts.
- Expand an understanding of the dynamics of collaborative governance as CGRs develop and change over time.

By aggregating up across case studies, research can develop more generalizable insights about collaborative dynamics, how collaborative governance regimes evolve over time, and how these efforts influence environmental management. This special issue extends the Database through the contribution of new case studies from 44 to 58. Several papers also apply comparative methodologies to study collaborative dynamics in environmental management.

This introductory article provides a summary of contributions of the 11 articles that comprise this special issue and draws out what we view as the significance of these contributions to collaborative governance debates.

## Overview of Papers in the Special Issue

This special issue solicited cases involving CGRs located in lesser studied political, geographic, and environmental contexts to explore the drivers and dynamics of collaborative governance regimes. Geographically, cases in the special issue describe collaborations on four continents: five in Europe (Germany, Italy, Sweden, UK); four in Asia (China, India, Vietnam); four in North America (USA); and one in South America (Brazil). Most collaborations are at the local or regional scale, but a few involve local-to-national collaborations (Bruun and Rubin 2022; Cristofoli et al. 2022) and one engages with supranational networks (Picavet et al. 2022). The cases included local contexts that were largely democratic societies as well as more authoritarian contexts. They collectively address diverse environmental topics such as water quality, habitat protection, energy, waste management, species conservation, and the social justice implications of environmental policy (Table 1). Given our focus on longitudinal dynamics, it is worth noting that the cases cover a variety of durations, spanning a minimum of three years (Cristofoli et al. 2022; one of the sage grouse partnerships, Taylor 2022), a median of 11 years, and a maximum of 52 years (one of the NEP partnerships, Imperial 2022). Finally, the cases illustrate the wide range of data and methodologies to examine collaborative governance. These include interviews (e.g., Zambrano-Gutiérrez et al. 2022), surveys (Bruun and Rubin 2022), focus groups (Lakshmisha and Thiel 2023), document analysis (Siddiki and Ambrose 2022), comparative case analysis (Imperial 2022, Taylor 2022, Zhou and Dai 2022), and qualitative comparative analysis (QCA) (Avoyan 2022).

**Table 1:** Overview of papers in this issue

Authors	Policy Domain	Case Topic	Analytical Focus
Avoyan 2022	Multiple	Comparative analysis of 16 environmental case studies	How collaborative governance shapes innovative solutions
Berthod et al. 2022	Energy	Five municipal and regional renewable energy transitions in Germany, Sweden, UK, and USA	Power dynamics shaping inclusion or restriction in policy goals and membership
Bruun and Rubin 2022	Water	Flood management in Vietnam	Formation and performance of collaboration in authoritarian settings
Cristofoli et al. 2022	Water	Flood management in Venice, Italy	How stakeholders overcome conflict and stalemate
Imperial 2022	Water	National Estuary Partnerships, USA	Formation, evolution, and death of collaborations
Lakshmisha and Thiel 2023	Water	Lake co-management in Bengaluru, India	Legitimacy, shared understanding, and resources as preconditions for collaboration
Picavet et al. 2022	Solid waste management	Climate resilient solid waste management infrastructure for Gangtok, India	How international networks shape local collaborative dynamics and outcomes
Siddiki and Ambrose 2022	Environmental justice	Environmental justice councils, USA	How representation and collaborative activities change over time
Taylor 2022	Endangered species	Sage grouse in Western USA	Accountability and trust in public-private partnerships
Zambrano-Gutiérrez et al. 2022	Agriculture, waste management	Urban agriculture and waste management in Florianópolis, Brazil	The role of individuals over time
Zhou and Dai 2022	Water	Water governance in Yangtze River Delta, China	How top-down interventions shape collaborative dynamics

Many of the CGRs highlighted in the special issue are focused on policy formation. For example, Siddiki and Ambrose (2022) study environmental justice councils as cases of collaborative governance, highlighting the role these groups play in identifying and designing policies that prevent marginalized communities from disproportionately bearing environmental harms. Others focus on the structure of CGRs and/or the processes needed to accomplish shared goals (i.e., implementation). For example, Cristofoli and colleagues (2022) examine how stalemates in collaborative environmental governance were managed to move towards a shared decision in defending Venice against floods. Likewise, Imperial’s (2022) examination of the lifecycles of watershed partnerships notes that the structures used to develop policy often need to be modified during the implementation phase.

## Contributions to Collaborative Governance Theory

The collection of articles featured in this special issue contribute to the development of collaborative governance theory and contribute to understanding different components of Ansell and Gash’s (2008) *Model of Collaborative Governance* and Emerson, et al.’s *Integrative Framework for Collaborative Governance* (2011). Further, they help inform the practice of collaborative governance while raising important questions to guide future research. Here we draw out the collective significance of the contributions to the Special Issue.

### Definitions Matter

At a fundamental level, the articles demonstrate the importance of using a broad definition of “collaborative governance”, such as the one proposed by Emerson and colleagues (2011, 2): “The processes and structures of public policy decision making and management that engage people constructively across the boundaries of public agencies, levels of government, and/or the public, private, and civic spheres in order to carry out a public purpose that could not otherwise be accomplished.” Having a shared definition of concepts is a fundamental building block for theory building. However, during the peer-review process for the articles featured in this issue, several reviewers questioned whether some of the cases constituted collaborative governance because they relied on narrower definitions such as that proffered by Ansell and Gash (2008, 544) that define collaborative governance as: “A governing arrangement where one or more *public agencies* directly engage non-state stakeholders in collective decision making that is formal, *consensus-oriented*, and deliberative and that aims to make or implement *public policy or manage public programs or assets* [*emphasis added*].” This definition is much narrower—perhaps seen as a subset of the collaborations in the Emerson et al. definition—and would eliminate several studies from this special issue.

We view the Emerson et al. definition of collaborative governance as conveying an instrumental perspective while the Ansell and Gash definition is structural. The former emphasizes the means by and through which collaboration occurs, whereas the latter emphasizes the structural form of collaboration. The advantage of viewing collaborative governance from an instrumental perspective is that it allows us to recognize that a wide range of drivers and institutional forms are possible where collaboration dynamics can be studied. For example, many of the watershed partnerships observed by Imperial (2022) would fail Ansell and Gash’s definitional test. While the partnerships formed as part of the EPA’s National Estuary Program (NEP) meet the test during the planning stage, the implementation structures that emerged for the Inland Bays and Tampa Bay (and later Tillamook Bay) do not clearly meet the Ansell and Gash definition. Yet, they are clearly collaborative partnerships. Likewise, collaborative approaches in non-democratic societies may not always operate in a consensus-oriented manner or involve non-state agencies, yet they can still provide important lessons about how different organizations or levels of government manage power-sharing and communication as they work together. Working in the case of Vietnam, Bruun and Rubin (2022) coin the term “captured collaboration” to highlight that despite there being clear interdependence both vertically and horizontally, collaborations were dominated by the central state both in function and process. Other terms have been applied in the collection of papers in this SI or elsewhere such as co-management (e.g. Lakshmisha and Thiel 2023) or polycentric governance which demonstrate conceptual overlapping with notions of collaborative governance. Overall, using a broad, institutionally oriented understanding of interorganizational relationships in debates and theory development about governance, rather than maintain a stickling for any one term, helps to facilitate learning from and between diverse contexts.

### Drivers of Collaborative Governance

This collection of articles also improves our understanding of the drivers of collaborative governance. The literature provides a variety of reasons that collaborative governance regimes are established (Imperial et al. 2018). They can be initiated by government, citizens groups and stakeholders, or a mix of both (Moore and Koontz 2003; Ulibarri et al. 2020). Partnerships can self-organize to address a common problem, provide a service, or accomplish a task (Huxham and Vangen 2000; Huxham 2003). Some are designed deliberately and reflect the *intentionality* resulting from the shared goals of founding members (Katz and Gartner 1988). Others are emergent and take shape as participants grapple with different challenges (Head 2008; Imperial et al. 2016). Government agencies, funders, or other ‘top-down’ forces can also encourage or compel members to participate in a collaborative governance regime (Moore and Koontz 2003; Emerson and Nabatchi 2015a). External organizations can also serve as a convenor and catalyze initiation of a collaborative process (Koontz et al. 2004; Ansell and Gash 2018).

Several papers in this special issue provide examples of self-initiated collaborative governance (Berthod et al. 2022; Lakshmisha and Thiel 2023; Taylor 2022). For example, worried that a proposed regulation would impact their operations, ranchers in Oregon, USA reached out to federal, state, and local government agencies to negotiate conservation agreements that would protect species while maintaining ranching operations (Taylor 2022). Five cases were externally driven, resulting from a mandate or grant-in-aid program that imposed the structural formation of a collaborative partnership (Bruun and Rubin 2022; Cristofoli et al. 2022; Siddiki and Ambrose 2022; Zhou and Dai 2022; Idaho case in Taylor 2022). There were also three cases convened by an external facilitator (Picavet et al. 2022; Zambrano-Gutiérrez et al 2022; UK case in Berthod et al. 2022). For example, Picavet and colleagues (2022) examine the way that ICLEI – a transnational network of cities – helped a municipality in India develop a collaboration to manage waste in a more climate friendly way.

The papers do not suggest a clear correlation between specific contexts and which type of initiation style is used. For instance, Imperial’s (2022) analysis of 31 watershed partnerships concluded that about half (16) of the partnerships (or changes to partnership structures) were self-initiated while the rest were externally driven, despite being located within the same four watersheds and all aiming to solve the same general problem (poor water quality). Likewise, the renewable energy collaborations studied in Berthod et al. (2022) include both self-initiated and externally convened collaborations.

These articles show that many collaborative governance regimes are born from complex environmental problems that require the mobilization of a collection of governmental actors and stakeholders. However, they clearly can be mobilized in different ways. More work needs to be done to better understand how differences in these drivers shape the formation of new CGRs or the frequent reorientations and recreations that occur over the course of their lifecycles (Imperial 2022; Imperial et al. 2016; Ulibarri et al. 2020). It is also unclear how a CGR’s initiation type is linked to developmental processes and collaborative dynamics. An earlier analysis by Ulibarri and colleagues (2020) of 21 collaborative partnerships suggests that self-initiated processes have a more deliberative, shared decision-making process, higher leadership, and provided more accountability. However, the renewable energy cases examined by Berthod and colleagues (2022) were almost entirely self-initiated, yet their early activation phase was very turbulent and several of the cases never matured into more stable or effective collaborations. Understanding how initiation type shapes longitudinal dynamics can provide important insight for higher level authorities who use collaborative governance as a policy tool to externally drive the creation of new collaborative governance regimes (Ansell and Gash 2018; Imperial 2022; Scott and Thomas 2017).

### Lifecycle Dynamics

Several papers in the special issue extend the four-stage lifecycle model proposed by Imperial and his colleagues (Imperial 2022; Imperial et al. 2016; Ulibarri et al. 2020), which posits that collaborations begin in a turbulent activation stage, slowly become a more well-defined collectivity, become institutionalized as roles and activities become more stable, and lastly undergo varied trajectories of stability, change, or decline. In one paper that directly adopts this model, Zhou and Dai (2022) look at the activation through institutionalization phases of a water management collaboration in China (a transition that took approximately 17 years). Specifically, they study the tools that higher-level authorities use to shape the collaboration as it evolves and find a gradual transition from more hands-on tools to hands-off, less intrusive forms of intervention.

In general, the papers in the special issue find that collaborations are far more dynamic than the original four-stage model conceptualized. Imperial’s (2022) study of 31 partnerships in four watersheds highlights the myriad of developmental trajectories that occur as the developmental process converges on a stable structure and highlights the changes (reorientations and recreations) that often occur. Siddiki and Graham’s (2022) study of environmental justice councils shows that collaborations engage, at any given time, in activities characteristic of multiple stages. As such, their study suggests that classification of collaborations according to lifecycle stage should be based on which kind of activity(ies) a collaboration is dominantly engaging in at any given point, rather than based on the presence or absence thereof.

Further work is needed to better understand how participants collectively select the rules that give rise to stable structures (i.e., CGRs) and what these rule configurations are. We also need to better understand the institutional dynamics at the heart of the lifecycle model, to understand how rules structure the principled engagement, create the shared motivation, and enable the capacity for joint action that is at the heart of the Integrative Framework for Collaborative Governance (Emerson et al. 2011). We also remain a long way from understanding whether certain partnership “structures” are better for deploying certain collaborative “tools” or achieving certain purposes (Prentice et al. 2019).

### Individuals and their Role in Collaborative Governance

Attracting, embracing, and supporting the ‘right’ participants and determining when they should enter (or exit) a collaboration is a challenge that never dissipates (Agranoff and McGuire 2001; Johnston et al. 2010, 703; O’Leary et al. 2012; Saz-Carranza and Ospina 2010; Vangen and Huxham 2003). Much of the collaboration literature assumes that individuals represent specific organizations or stakeholder groups interests, overlooking the specific set of individual skills, personal interests/values/motivations, and previous experience/knowledge held by the individuals. There are several papers in this special issue that view collaborative processes from an individual perspective, highlighting the vital role of leaders and other actors in shaping the trajectory and performance of CGRs.

Individuals had both positive and negative influence on collaboration. For example, in studying the development of flood management regimes in Venice, the authors highlight the “orchestrational” work of the Mayor of Venice in facilitating stakeholder convergence and the breakthrough of a decision-making stalemate (Cristofoli et al. 2022). In the case of waste management and urban agriculture collaboration in Brazil, Zambrano-Gutiérrez and coauthors (2022) highlight dynamism in the roles filled by individual participants over time and in the organizations which they represent. They find that over time, individuals switched roles within and between organizations. These moves changed the individual’s motivation to collaborate and over time led to the decline of the collaboration.

### Other Factors Influencing Collaborative Dynamics

The papers in the special issue highlight two additional factors that influence collaboration dynamics, both of which are included as part of the coding scheme for the Database: political dynamics and accountability. Several papers discuss the ways political dynamics (both internal and external) shape collaborative dynamics. For instance, Berthod and colleagues (2022) describe several cases where powerful government actors “used institutional rules to co-opt and shut down the [collaborative] movement”, limiting the scope of what was attempted and who was involved and conflicting with the broader aims of energy democracy that was driving the collaborations. Likewise, Bruun and Rubin (2022) study the development of a water management collaboration in Vietnam. The collaboration was formed in response to international pressures for more participatory governance, yet in operation was highly constrained by the ruling Communist Party of Vietnam.

Accountability is another factor featured in the Database’s coding scheme. Accountability is a complicated aspect of collaboration dynamics. On the one hand, both formal (e.g., written agreements, shared plans) and informal (e.g., social norms, peer pressure) accountability mechanisms can be powerful motivators for individuals/organizations to participate and contribute to collaborative processes. However, too much accountability can serve as a demotivator and inhibit participation (Imperial 2005b). The paper by Taylor (2022) on public-private partnerships to protect the endangered sage grouse highlights the complex interplay between trust and accountability. In her case, government agencies were concerned with the need to maintain legal accountability (i.e., to their mandate under the Endangered Species Act) to avoid lawsuits, and this limited the flexibility in what they could offer partners in the collaboration. Being accountable to the law but not to their partners ultimately undermined the other participants’ trust in the collaboration. The Lakshmisha and Thiel (2023) paper sheds light on mechanisms for developing internal and external legitimacy, highlighting the interplay between government actors as custodians of the participatory process and third-sector organizations as bringing together heterogeneous communities to support shared problem definition.

### Understanding the Outcomes of Collaborative Governance Regimes

The papers in this special issue were largely focused on the drivers and dynamics at the heart of the collaborative governance, rather than on the outcomes of those processes. The exception is the comparative analysis by Avoyan (2022), which evaluated the different conditions under which collaborations achieved innovative outcomes. She found that “functional institutional design coupled with either considerable knowledge sharing or productive collaborative process and effective collaborative leadership may be sufficient to reach the outcome: innovative solutions” (Avoyan 2022, 11).

That said, the papers do highlight several challenges associated with evaluating the outcomes of collaborative processes. There are numerous ways to evaluate the outcomes of a CGR. In a meta-review of collaborative conservation, Koontz et al. (2020) highlighted diverse criteria used by researchers to measure outputs and impacts. These generally grouped into two categories: the first relating to ecological outputs and impacts such as conservation or use outcomes, and the second describing different social consequences, like behavioral change or social capital building. However, which of these outcomes is desirable depends on the perspective used to judge whether the collaborative process was “effective” or “successful” (Emerson and Nabatchi 2015b; Koontz and Thomas 2006). Because collaborations are initiated to address some sort of “wicked” problem (Baird et al. 2016) that cannot be solved by anyone actor working alone. This means judgements can be framed from multiple perspectives:

- *Perspective 1*: Judgements are framed in terms of making a difference in terms of the problems that motivated joint action.
- *Perspective 2*: Judgements are framed in terms of the expectations of external actors (higher level authorities, funders, stakeholders, etc.) who have their own view of what the CGR should accomplish.
- *Perspective 3*: Judgements are framed in terms of what the CGR sets out to accomplish, which may or may not coincide with the views of external actors.

Perspective 1’s traditional notions of “impacts” or “effectiveness” implies that the underlying purpose of a CGR is oriented around policy outcomes. This need not be the case. Collaborative governance regimes are formed for many purposes and can be used to enhance coordination, shared policy making, prioritizing actions or funding decisions, and accomplish projects (Imperial 2005a). As a result, some researchers use the concept of a healthy and useful life in place of terms like “success” or “performance” (Imperial et al. 2016; Imperial 2022). However, this change in orientation still produces conflicting views of “health” or “success” when viewed from perspectives 2 and 3. Imperial (2022) identifies several examples where watershed partnerships were relatively healthy when viewed from the partnership’s point of view (perspective 3) but some external stakeholders were disappointed with the same partnerships because they failed to make a tangible difference in watershed problems that motivated the partnership’s original initiation (perspectives 1 and 2). This suggests the need to explicitly evaluate success for whom.

A second challenge is the timing of when evaluative judgements occur. The four-stage lifecycle model argues that CGRs create *value* in different ways as they develop (Voets et al. 2008; Mandell and Keast 2008). What is necessary to achieve healthy developmental processes when they are forming is often quite different from what is needed in mature networks (Genskow and Born 2006, 56; Ulibarri et al. 2020). This likely explains the contradictory advice that is often found the collaborative environmental management literature (e.g., Leach and Pelkey 2001). Researchers examining “successful” efforts during early developmental stages may identify factors like facilitation, leadership, building relationships, and trust as critical factors while analyses of mature networks may highlight the importance of institutionalizing network structures and secure resource streams. Accordingly, evaluative judgements need to account for the stage of the developmental process.

Given that dynamic nature of collaborative governance, more work is needed to understand how to conceptualize the outcomes of CGRs. The Institutional Analysis and Development (IAD) framework proposed by Ostrom and her colleagues (Ostrom 1990, 1999, 2005; Ostrom et al. 1993; Imperial and Yandle 2005; Imperial 1999) may offer some guidance. The IAD framework advocates evaluating using multiple criteria to assess the performance of institutional arrangements and really focuses on the transaction costs associated with developing and administering these arrangements. However, institutional performance may or may not be connected to outcomes (Imperial and Yandle 2005). More research is needed to understand not only how to assess the performance of CGRs, but to also understand how the outcomes of the collaborative processes should be judged over time.

## Summary and Conclusions

The collection of articles in this special issue demonstrates that collaborative governance serves a variety of purposes and is used around the world to address complex environmental problems. Understanding the drivers and dynamics of collaborative governance is critical to theory building and providing useful advice to practitioners. The objective of this special issue is to bring together a collection of new case studies that are part of the Collaborative Governance Case Database and then use small-n and medium-n comparative studies to further theory building. By using a broad definition of collaborative governance proposed by Emerson and colleagues (2011, 2), this collection of articles reveals the wide range of drivers and institutional structures that can be crafted to enable collaborative governance. The articles also demonstrate that collaborations can be self-initiated, externally driven by mandates or incentives through grants, or convened by external facilitators. Improving our understanding of these drivers is critical to improving our ability to effectively initiate new collaborative efforts.

Individually and collectively, the papers make several important contributions to theory building and highlight areas where further research is needed. When viewed longitudinally, collaborations are far more dynamic and follow a wider range of developmental trajectories than were originally depicted in the four-stage lifecycle model proposed by Imperial and his colleagues (2016). More research is needed to better understand and manage the turbulent period surrounding the initiation of new collaborations. Moreover, improving our understanding of the institutional dynamics that underpin the lifecycle model will further enhance theory building and allow practitioners to develop structures that enhance the capacity for joint action.

Two of the studies in the special issue also contribute to our understanding of individuals and the roles they play in collaborative governance. This is an often-neglected area of collaboration research because many researchers assume that individuals simply represent the interests of the organization they represent. However, the studies here note that individuals have both positive and negative influences on collaborations. Individuals can play different roles within the CGR and within their organization, which can increase or decrease their motivation to collaborate.

The papers highlight two additional factors that shape collaborative dynamics. First, many of the papers note that political dynamics (both internal and external) are an important part of collaborative governance. They can inhibit or facilitate partnership formation and influence developmental dynamics in profound ways. Second, accountability is an important, yet complicated aspect of the dynamics of collaborative processes. Accountability can motivate participation and contributions to collaborative processes while too much accountability may serve as a disincentive to collaborate. Moreover, there is a complex interaction between trust and accountability. Being accountable to the law and external authorities but not their partners can undermine trust in the collaboration.

The role of individuals, political dynamics, and accountability may become increasingly important considerations for collaboration in a polarized world. Instead of striving for representation of all concerned stakeholders, conveners and facilitators may need to more strategically consider the specific personalities and motivations that will work together in a hyper-politicized (and potentially violent) context. For example, it may be necessary to first support building a “radical center” that can develop trust and establish ground rules, and then overtime grow to include more extreme voices that would agree to work within the established collaboration.^2^

While the papers in the special issue primarily focused on the drivers and dynamics of collaborative governance rather than the outcomes, the collection of papers highlights the challenges associated with evaluating the outcomes of collaborative processes. Judgements can be framed from different perspectives, which each present different methodological challenges. Given our more nuanced understanding of how collaborations develop over time, measures of “success” are likely to vary over the course of the developmental process and the factors that support success are likely to change as collaborations move through the developmental process. Accordingly, more research is needed to understand not only how to assess the performance of collaborative governance regimes, but to understand how to judge performance during the developmental process. Where possible, research will benefit from multi-case studies and cross-case comparisons, including across socioeconomic and political differences, to progress integrative theory building that combines insights from often disparate streams of research, for example from collaborative conservation, natural resource co-management, polycentric governance, and collaborative governance in environmental management.

The special issue lastly demonstrates the importance of resources such as the Collaborative Governance Case Database. This collection of articles highlights the theory-building potential of both small-n and medium-n comparative studies. Our hope is that researchers will consider coding and contributing their existing case studies or utilize the common coding framework in future research so that collaboration research can move to the next generation of collaborative governance theory building. Vital to this project is expanding the Database and other collective research endeavors to engage and integrate understandings about the drivers, dynamics and outcomes of collaboration from diverse contexts.

We hope this special issue will spur continued work to expand the geographies and contexts where we study collaborative environmental governance. It is important to note that research on forms of collaboration outside of Europe and North America is extensive and rich, but specific attention is required to generate the necessary dialogue to facilitate cross-contextual learning. Access to the Database is an important tool to facilitate comparison, and this SI contributes knowledge to collaborative governance debates from diverse contexts like Brazil and India. While exchange between some of the contributing authors occurred through a seminar in 2021, and many of the papers engage with other cases from the Database, we recognize more is needed to bring into dialogue regions with significant socioeconomic and political differences in a way that supports deep exchange and learning. Many barriers hamper this project, from research funding models that exclude or restrict international collaboration (e.g., grants often place unreasonable reporting and financing controls on international collaborators or they directly prohibit international co-investigators receiving funding) to the limitations of individuals’ or research groups’ theoretical and cognitive orientation based on the trajectory of their work. More effort is required, in particular on the part of privileged Global North institutions and scholars, to facilitate collaborative and comparative research on equitable grounds to illuminate important shared trends and unique practices that can progress collaborative governance in environmental management. Decolonial, feminist and indigenous theorists may offer important frameworks to guide such work.

## Footnotes

1 Importantly, the cases in the Database are not representative of all instances of collaborative governance. As they are voluntarily submitted by authors, there is bias in coverage—which partially motivated our effort to include less-studied geographic and sectoral contexts.

2 Thank you to one of our anonymous reviewers for this insight.
